# Genomic Analysis and Advanced Mining of *Photorhabdus* Isolates Reveal Cryptic Polyketide Synthase Clusters and Potent Anti-Staphylococcal Activity

**DOI:** 10.3390/microorganisms14102378

**Published:** 2026-10-16

**Authors:** Wipanee Meesil, Yaowapa Sikhaophong, Kasem Chanjitwiriya, Yeunyong Kantanet, Liam K. R. Sharkey, Sacha J. Pidot, Apichat Vitta, Aunchalee Thanwisai

**Affiliations:** 1 Department of Microbiology and Parasitology, Faculty of Medical Science, Naresuan University, Phitsanulok 65000, Thailand; wipaneem.work@gmail.com (W.M.); yaowapas67@nu.ac.th (Y.S.); kasemc@nu.ac.th (K.C.); apichatv@nu.ac.th (A.V.); 2 Siriraj Cancer Center, Faculty of Medicine Siriraj Hospital, Mahidol University, Bangkok 10700, Thailand; 3 Center for Animal Research, Naresuan University, Phitsanulok 65000, Thailand; 4 The School of Information and Communication Technology, University of Phayao, Phayao 56000, Thailand; yeunyong.ka@up.ac.th; 5 Department of Microbiology and Immunology, Doherty Institute, Melbourne 3000, Australia; liam.sharkey@unimelb.edu.au (L.K.R.S.); sacha.pidot@unimelb.edu.au (S.J.P.)

**Keywords:** *Photorhabdus*, biosynthetic gene cluster, genome mining, deep learning

## Abstract

This study investigates the genomic and antibacterial potential of five *Photorhabdus* isolates recovered from *Heterorhabditis* sp. entomopathogenic nematodes collected from soil samples in northern Thailand (Kamphaengphet and Phitsanulok provinces). Whole-genome sequencing combining Illumina short-read and Oxford Nanopore long-read platforms, followed by phylogenetic analysis, digital DNA–DNA hybridization (dDDH) and average nucleotide identity (ANI) calculations, classified strain KLK8.1 as *Photorhabdus hainanensis* and strains KLK18.5, NMP12.2, NMP13.3, and NMP13.5 as *Photorhabdus akhurstii*. Genome mining using antiSMASH v8.0 and DeepBGC v0.1.23 identified 134 biosynthetic gene clusters (BGCs) across the five genomes, including conserved clusters associated with symbiosis and a substantial proportion (~60%) of clusters lacking characterized homologs. Deep-learning-assisted mining further identified rare, highly reducing type II polyketide synthase (HR-T2PKS) clusters, which were predicted to be associated with polyene biosynthetic pathways. Antibacterial profiling by disc diffusion and minimum inhibitory concentration assays revealed that the crude extracts exhibited potent activity against *Staphylococcus aureus* and selective activity against *Acinetobacter baumannii*. These findings highlight the unexplored biosynthetic capacity of Thai *Photorhabdus* isolates and support their potential as valuable sources for discovering novel bioactive compounds.

## 1. Introduction

Given the rising threat of antimicrobial resistance, exploring new natural products from underexplored microorganisms is increasingly necessary for discovering novel bioactive compounds [1,2]. The genus *Photorhabdus* (family Morganellaceae, order Enterobacterales) comprises Gram-negative, facultatively anaerobic, bioluminescent, rod-shaped bacteria currently encompassing more than 20 validly described species and is recognized as a promising source of diverse secondary metabolites [3,4]. These bacteria exhibit a dual lifestyle, alternating between mutualistic symbiosis with entomopathogenic nematodes of the genus *Heterorhabditis* and pathogenesis in insect larvae living in the soil, such as tobacco hornworm (*Manduca sexta*), diamondback moth (*Plutella xylostella*), and various caterpillars [5,6]. Adapting to these distinct ecological niches requires specialized chemical strategies, driving *Photorhabdus* to produce a broad repertoire of secondary metabolites for host interactions, microbial competition, and pathogenicity [5]. Secondary metabolites produced by these bacteria span multiple chemical classes. Well-characterized examples include nonribosomal peptides (NRPs) such as gamexpeptides and kolossin, which exhibit antibacterial, antifungal, and insecticidal activities [7,8]. Polyketide-derived metabolites include anthraquinone pigments and stilbene compounds with broad-spectrum antimicrobial and immunosuppressive properties [9]. Hybrid NRP–polyketide scaffolds such as luminmycins expand the structural diversity further [10]. Ribosomally synthesized and post-translationally modified peptides (RiPPs), siderophores (e.g., photobactin, putrebactin), terpene-derived carotenoids, and protease inhibitors such as leupeptin are also well represented [4,11,12]. Beyond direct antimicrobial effects, several *Photorhabdus* metabolites, including the insecticidal toxin complexes (Tc proteins) and the Mcf (“makes caterpillars floppy”) toxin, function in host killing and immune evasion [13].

Many of these secondary metabolites are encoded by biosynthetic gene clusters (BGCs), organized genomic units that contain the enzymatic machinery required for production [14]. The rapid expansion of genomic data has transformed natural-product discovery by enabling the identification of biosynthetic potential directly from genome sequences [15]. However, a major challenge is that a substantial proportion of predicted BGCs remain uncharacterized or show limited similarity to known biosynthetic pathways, representing a potentially important source of cryptic natural products [14]. Rule-based genome-mining platforms such as antiSMASH have been instrumental in detecting and classifying canonical BGCs [16]. Nevertheless, approaches that rely primarily on predefined biosynthetic signatures or homology may have limited ability to recognize highly divergent or atypical clusters. Complementary computational approaches, including machine-learning and deep-learning methods, may therefore facilitate the detection of biosynthetic regions that are difficult to identify using conventional approaches [17]. Deep-learning-assisted genome mining provides an additional strategy for exploring poorly characterized biosynthetic regions. DeepBGC, for example, uses a bidirectional long short-term memory (BiLSTM) neural network to identify genomic regions with features characteristic of BGCs and to predict broad biosynthetic classes [18]. Combining such predictive approaches with established rule-based tools can provide complementary information and may expand the range of BGCs considered for downstream investigation.

This is particularly relevant for *Photorhabdus*, for which comparative genomic studies have revealed diverse biosynthetic repertoires [3,4]. The biosynthetic potential of newly sequenced *Photorhabdus* isolates from underexplored environments therefore remains an important area for investigation. Addressing these gaps is important both for understanding the intraspecific chemical diversity of the genus and for identifying strains with high potential for natural-product discovery. Therefore, in this study, we investigated five newly sequenced *Photorhabdus* isolates obtained from entomopathogenic nematodes (*Heterorhabditis* sp.) collected in Thailand. We first characterized their taxonomic relationships using whole-genome sequencing, phylogenomic reconstruction, digital DNA–DNA hybridization (dDDH), and average nucleotide identity (ANI) analyses. We then profiled their biosynthetic potential in silico using complementary rule-based (antiSMASH v8.0) and deep-learning-assisted (DeepBGC v0.1.23) genome-mining approaches. Finally, we assessed the antibacterial activity of whole-cell suspensions and ethyl acetate crude extracts against a panel of antibiotic-resistant bacterial pathogens. By integrating genomic analysis, multi-tool genome mining, and phenotypic screening, this study aimed to reveal underexplored biosynthetic features unique to Thai *Photorhabdus* isolates and to prioritize strains for future investigation as potential sources of novel antimicrobial metabolites.

## 2. Materials and Methods

### 2.1. Data Collection and Sequencing

Five *Photorhabdus* isolates (Table 1) were selected for genome sequencing based on their antibacterial activity screening results and were obtained from Associate Professor Dr. Apichat Vitta, Naresuan University, Thailand. Strains KLK8.1 and KLK18.5 originated from the Konglan District, Kamphaeng Phet Province, whereas NMP12.2, NMP13.3, and NMP13.5 were collected from the Noen Maprang District, Phitsanulok Province, in 2023. All isolates were recovered from *Heterorhabditis* sp. entomopathogenic nematodes collected from soil as previously described [19]. Briefly, EPNs were isolated using the *Galleria mellonella* baiting method, and symbiotic bacteria were recovered from infected larvae and cultured on nutrient agar supplemented with bromothymol blue and triphenyl-2,3,5-tetrazolium chloride (NBTA) medium at room temperature for 3–4 days. The isolates were preliminarily identified as *Photorhabdus* based on characteristic light- to dark-green colonies on NBTA and *recA* gene sequence analysis. For whole-genome sequencing, a single colony of each bacterial strain was inoculated into 5 mL of LB broth (Sigma-Aldrich, Buchs, Switzerland) and incubated at 28 °C with agitation at 200 rpm for 24 h [20]. Genomic DNA was extracted from cultured samples using the DNeasy Blood and Tissue Kit (Qiagen, Hilden, Germany) according to the manufacturer’s instructions. For all isolates, whole-genome sequencing (WGS) libraries were prepared for both Illumina short-read and Oxford Nanopore Technologies (ONT) long-read sequencing. Illumina libraries were constructed using the Illumina DNA Prep kit and sequenced on the NextSeq 500 platform (Illumina, San Diego, CA, USA) to generate 150 bp paired-end reads. ONT libraries were prepared using the SQK-NBD114-96 and SQK-RBK114-96 kits (Oxford Nanopore Technologies, Oxford, UK) and sequenced on FLO-MIN106D (R9.4.1) flow cells using a GridION Mk1 platform. Basecalling of ONT reads was performed using Guppy v6.0 [21]. All sequencing procedures were conducted at the Doherty Applied Microbial Genomics facility, The University of Melbourne, Australia.

### 2.2. Data Quality Control and Preprocessing

Raw sequencing data were subjected to quality control (QC) utilizing FastQC v0.12.0 [22] to assess read quality metrics. QC reports across all samples were aggregated and summarized using MultiQC v1.33 [23]. Illumina paired-end reads were preprocessed using Trimmomatic v0.39 [24] to remove universal and library-specific adapter sequences, redundant sequences, and low-quality bases. Trimming was executed using a Phred quality score cutoff of Q30, and reads shorter than 50 bp post-trimming were discarded to ensure high sequence fidelity. ONT long reads were filtered using Filtlong v0.2.1 [25], retaining the top 90% of reads based on quality scores and applying a minimum read length threshold of 1000 bp to eliminate short and low-quality sequences.

### 2.3. Genome Assembly, Polishing, and Annotation

Hybrid genome assembly was performed using Unicycler v0.5.0 [26], which integrated the trimmed Illumina short reads with the filtered ONT long reads to generate contiguous draft assemblies. To maximize base-level accuracy, assembly polishing was subsequently carried out. Illumina reads were aligned to the draft assemblies using BWA-MEM v0.7.17 [27], and the resulting alignment files were sorted and indexed utilizing SAMtools v1.22.1 [28]. Polishing was executed with Pilon v1.24 [29] to systematically correct base substitutions, small insertions/deletions, and local misassemblies. Overall assembly quality was evaluated using QUAST v5.2.0 [30] to assess metrics such as N50, total assembly length, and contig number. Gene prediction and functional annotation of the finalized genome assemblies were conducted using Prokka v1.14.6 [31] under default parameters. Genome functional annotation was then performed using eggNOG-mapper v2 [32].

### 2.4. Taxonomic and Phylogenomic Analysis

Whole-genome-based taxonomic analysis was performed using the Type (Strain) Genome Server (TYGS) [33], available at https://tygs.dsmz.de (accessed on 25 June 2025). Closely related type strains were identified based on genome comparisons and 16S rRNA gene sequence analysis. Genome-wide intergenomic distances were calculated using Genome BLAST Distance Phylogeny (GBDP), and digital DNA–DNA hybridization (dDDH) values were calculated using GGDC 4.0 [34]. Phylogenomic relationships were reconstructed from the resulting GBDP intergenomic distance matrix using FASTME 2.1.6.1 [35], with 100 pseudo-bootstrap replicates used to assess branch support. The resulting tree was midpoint-rooted and visualized using PhyD3 [36]. Species and subspecies delineation was performed using 70% and 79% dDDH thresholds, respectively. In addition, the polished genome assemblies were subjected to average nucleotide identity (ANI) analysis using FastANI v1.34 [37], with a 95% ANI threshold applied for species-level assignment.

### 2.5. Biosynthetic Gene Cluster (BGC) Prediction

To comprehensively map the secondary metabolome of the *Photorhabdus* isolates, genome mining for biosynthetic gene clusters (BGCs) was conducted using a dual in silico approach. Initially, the genomes were analyzed using antiSMASH v8.0 [38] under default parameters. This rule-based tool detects and classifies BGCs (e.g., polyketide synthases [PKS], nonribosomal peptide synthetases [NRPS], and ribosomally synthesized and post-translationally modified peptides [RiPPs]) by comparing genomic regions against a curated database of known clusters. To complement these rule-based predictions and capture highly divergent or atypical clusters that evade canonical signatures, the genomes were subsequently queried using DeepBGC v0.1.23 [18]. DeepBGC utilizes a bidirectional long short-term memory (BiLSTM) neural network to predict BGC boundaries and assign functional classes. Genomic FASTA files were processed with DeepBGC using default parameters. The BGCs predicted by both antiSMASH and DeepBGC were cross-examined to identify overlapping and unique clusters. Finally, to validate automated confidence scores, manual structural verification was performed. The domain and module architectures of core biosynthetic enzymes were visually inspected and compared against reference clusters in the MIBiG database [20,39].

### 2.6. Preparation of Extracts and Antibacterial Activity Assay

The antibacterial activity of the *Photorhabdus* isolates was evaluated using both whole-cell suspensions and ethyl acetate crude extracts, adapted from previously described methods [40]. Briefly, a single colony of each isolate was inoculated into 500 mL of LB broth and incubated at room temperature with continuous shaking at 180 rpm for 48 h. The resulting whole culture was extracted with 1000 mL of ethyl acetate. The organic phase was collected and concentrated to dryness via rotary evaporation at 40 °C to yield the crude extract.

Antibacterial screening was performed utilizing the disk diffusion assay against a broad panel of clinically relevant bacterial pathogens. Gram-negative strains included extensively drug-resistant (XDR) *Acinetobacter baumannii* (AB320, AB321, AB322, and AB324); *Escherichia coli* ATCC35218, multidrug-resistant (MDR) *E. coli* PB30, ESBL-producing MDR *E. coli* PB1, and ESBL/carbapenem-resistant (ESBL + CRE) *E. coli* PB231; *Klebsiella pneumoniae* ATCC700603, ESBL-producing MDR *K. pneumoniae* PB5, and ESBL + CRE *K. pneumoniae* PB21. Gram-positive strains included *Staphylococcus aureus* ATCC20475 and methicillin-resistant *S. aureus* (MRSA) strains PB36 and PB57.

Pathogen suspensions were adjusted to a final concentration of 10^8^ CFU/mL and uniformly swabbed onto Mueller–Hinton agar (MHA) plates (Oxoid Ltd., Hampshire, UK). Sterile paper discs impregnated with 10 µL of either the whole-cell suspension or the crude extract (reconstituted at 100 mg/mL in DMSO) were applied to the inoculated agar.

Standard antibiotic discs appropriate for each specific test organism were utilized as positive controls, whereas DMSO-only discs served as negative controls. Following incubation at 37 °C for 24 h, antibacterial activity was quantified by measuring the diameter of the clear zones of inhibition. Isolates exhibiting strong inhibitory activity were selected for further quantitative evaluation. The minimum inhibitory concentration (MIC) and minimum bactericidal concentration (MBC) values were determined using the standard broth microdilution method with two-fold serial dilutions. All measurements were performed in three independent experiments. MIC and MBC values within the tested concentration range were summarized as arithmetic means ± sample standard deviations (SD). The mean and sample SD were calculated using Python 3.12 with NumPy 2.0.2. Values exceeding the highest tested concentration were reported as >50 mg/mL. The figures were prepared using Python 3.12 with the Matplotlib 3.10.0, Seaborn 0.13.2, and NumPy 2.0.2 packages.

## 3. Results

### 3.1. Genome Characteristics and Taxonomic Classification

Five bacterial strains were previously isolated from entomopathogenic nematodes (EPNs) collected in Kamphaeng Phet and Phitsanulok provinces, Thailand, in 2023 (Table 1). To further characterize these isolates and determine their taxonomic identity, whole-genome sequencing (WGS) was performed, providing a genomic foundation for subsequent investigation of their bioactive potential. Draft genome assemblies were obtained for all five isolates, with genome sizes ranging from 5.52 to 5.85 Mb. The assemblies comprised 4–68 contigs, with N50 values ranging from 705,958 to 4,717,545 bp and GC contents of 42.81–43.25%. CheckM2 v1.1.0 analysis [41] estimated 100% completeness for all five genomes, with contamination levels ranging from 0.05% to 4.39%. NMP12.2 showed the most fragmented assembly and the highest estimated contamination, with 68 contigs, an N50 of 705,958 bp, and 4.39% contamination. No plasmids were detected in any of the five strains. Additional genome assembly statistics are provided in Supplementary Table S1.

**Figure 1:**
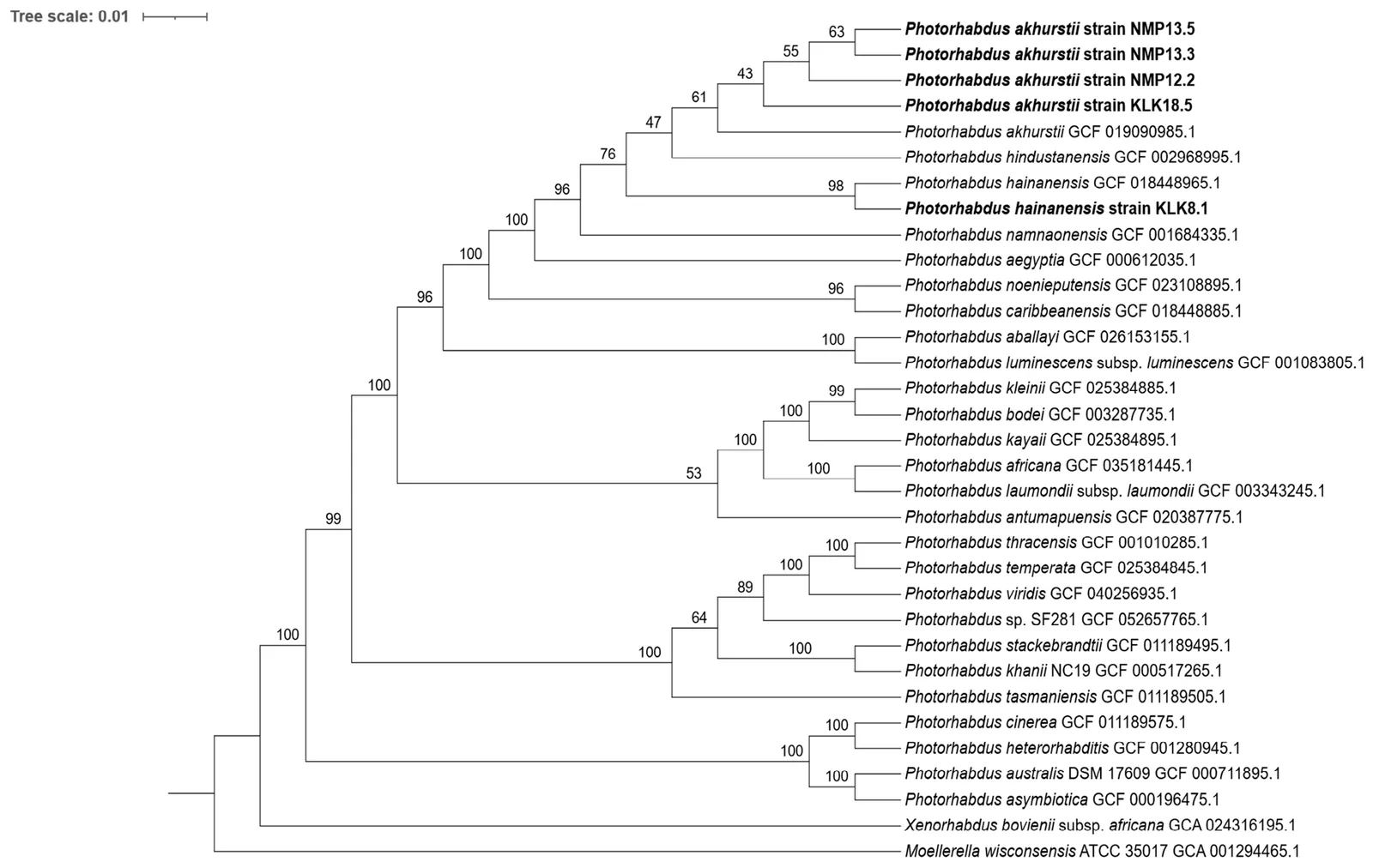
Phylogenomic tree of *Photorhabdus* strains reconstructed using the TYGS platform based on whole-genome sequences. Genome-wide intergenomic distances were calculated using Genome BLAST Distance Phylogeny (GBDP), and the tree was reconstructed using FASTME with 100 pseudo-bootstrap replicates. Pseudo-bootstrap support values (>50%) are shown at branch nodes. Newly sequenced isolates from this study are in bold. *Xenorhabdus bovienii* and *Moellerella wisconsensis* were included as outgroup taxa.

To investigate evolutionary relationships among the isolates, phylogenomic reconstruction was performed using the TYGS platform [33]. The resulting genome-based phylogeny resolved four major clades within the genus *Photorhabdus* (Figure 1). Strains KLK18.5, NMP12.2, NMP13.3, and NMP13.5 clustered within a well-supported monophyletic clade with the reference strain *P. akhurstii* DSM 15138, originally isolated from *H. indica*. In contrast, strain KLK8.1 grouped with established *P. hainanensis* reference genomes, forming a distinct lineage separate from the *P. akhurstii* clade. The corresponding dDDH values are provided in Supplementary Table S2.

ANI analysis further supported these genome-based taxonomic assignments. Strain KLK8.1 showed 97.75% ANI with *P. hainanensis* DSM 22397, whereas strains KLK18.5, NMP12.2, NMP13.3, and NMP13.5 showed ANI values of 97.86%, 97.87%, 97.76%, and 97.72%, respectively, with *P. akhurstii* DSM 15138 (Table 1). All values exceeded the 95% ANI threshold commonly used for species-level delineation. Taken together, the phylogenomic, dDDH, and ANI results supported the species-level classification of KLK8.1 as *P. hainanensis* and of KLK18.5, NMP12.2, NMP13.3, and NMP13.5 as *P. akhurstii*. Newly sequenced strains are highlighted in bold in Figure 1.

Additionally, COG-based functional annotations using eggNOG-mapper v2 [32] of all five genomes revealed broadly similar distributions across strains (Supplementary Figure S1). In all isolates, the largest functional category was S (Function unknown; 25.4–28.4%), followed by transcription (K; ~9.3–9.8%) and cell wall/membrane biogenesis (M; ~6.9–7.5%). Genes assigned to category Q (secondary metabolite biosynthesis, transport and catabolism) accounted for 2.9–3.3% of all CDS, providing functional annotation-level corroboration for the extensive biosynthetic gene cluster repertoire described below.

### 3.2. Diversity and Distribution of Biosynthetic Gene Clusters (BGCs)

**Figure 2:**
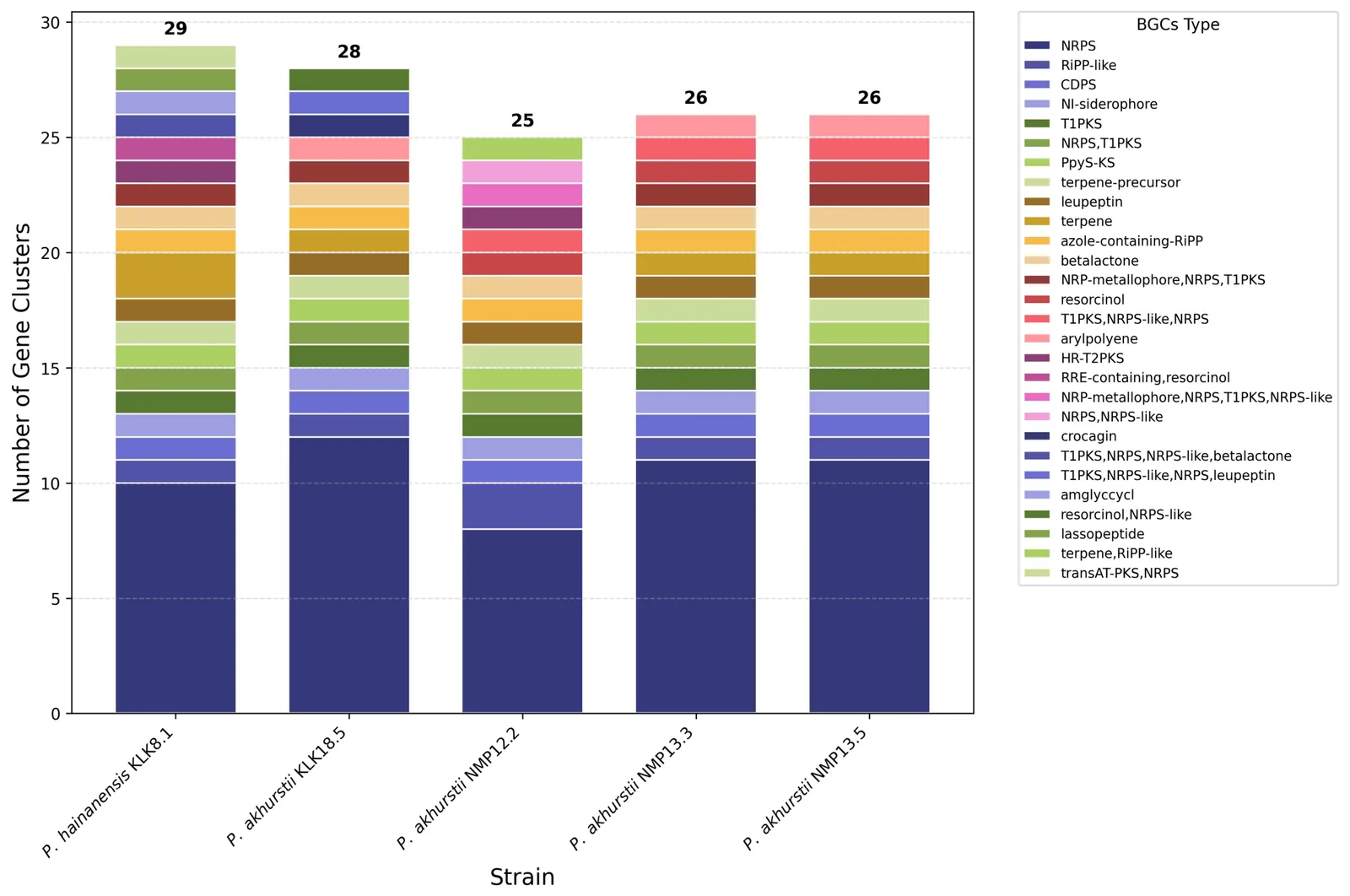
Biosynthetic gene cluster (BGC) profile of *Photorhabdus* isolates. A stacked bar chart illustrating the diversity and abundance of secondary metabolite gene clusters identified across *P. hainanensis* (strain KLK8.1) and *P. akhurstii* (strains KLK18.5, NMP12.2, NMP13.3, and NMP13.5). Colors represent distinct biosynthetic classes as predicted by antiSMASH and DeepBGC integrated analysis.

Comprehensive in silico genome mining revealed a dense and diverse biosynthetic landscape across all five isolates, with a total of 134 BGCs identified. The secondary metabolome was characterized by a high density of nonribosomal peptide synthetases (NRPSs), type I polyketide synthases (T1PKSs), and ribosomally synthesized and post-translationally modified peptides (RiPPs) (Figure 2; Table S3).

**Figure 3:**
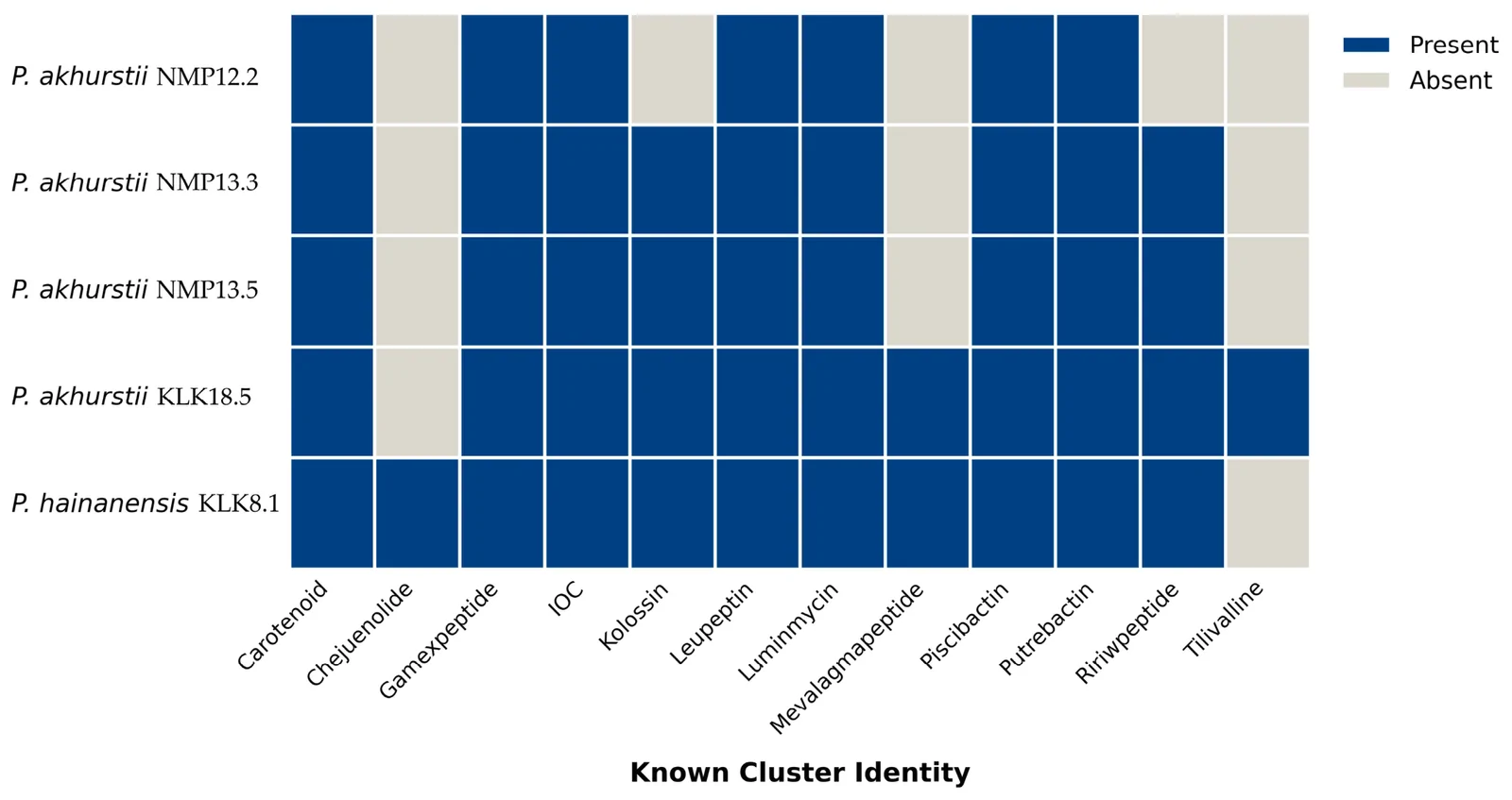
A plot displaying the distribution of known gene clusters across the five study isolates. Navy squares indicate the presence of a gene cluster, while gray squares indicate its absence. The analysis reveals a conserved core metabolite and strain-specific accessory traits, highlighting the chemical divergence between the two species.

Approximately 38.05% (51 BGCs) of the identified clusters showed high similarity to previously characterized pathways, representing a conserved core metabolome shared between the *P. hainanensis* and *P. akhurstii* strains (Figure 3). All five genomes contained the necessary biosynthetic machinery for the production of gamexpeptide [7], leupeptin [12], luminmycin [10], piscibactin [7], and putrebactin [11]. The BGC encoding kolossin [8] was also highly conserved, present in four of the five strains (absent only in *P. akhurstii* NMP12.2). Beyond this core metabolome, notable strain-specific variation was observed. *P. akhurstii* strain KLK18.5 was the only isolate predicted to produce tilivalline [42,43], a cytotoxin previously associated with *Klebsiella* pathogenicity. Similarly, *P. hainanensis* strain KLK8.1 uniquely harbored the BGC encoding chejuenolide [44], a distinct polyketide originally identified in a marine bacterium.

A substantial proportion of the identified clusters (approximately 60%) exhibited no significant similarity to known gene clusters, representing a vast reservoir of cryptic biosynthetic potential (Table S3). Among these, a taxlllaid-like NRPS cluster was identified in all five strains. Comparative analysis with the *Xenorhabdus indica* reference strain revealed architectural similarity to the taxlllaid A pathway, though sequence identity was moderate (64.84%).

All analyzed genomes also encoded uncharacterized azole-containing RiPP clusters structurally consistent with thiazole/oxazole-modified microcins (TOMMs). Furthermore, biosynthetic pathways for resorcinol and arylpolyene-like metabolites were universally present, alongside uncharacterized terpene-class BGCs and large, modular hybrid PKS–NRPS pathways.

Notably, rare highly reducing type II polyketide synthase (HR-T2PKS) clusters were identified in *P. hainanensis* strain KLK8.1 (Cluster 7) and *P. akhurstii* strain NMP12.2 (Cluster 22) (Table S3). These loci encode a specialized ketosynthase-chain length factor (KS-CLF) complex that is distinct from canonical aromatic PKSs. This architecture is associated with a biosynthetic process involving sequential reduction and dehydration steps that can generate linear polyene backbones [45]. The presence of these systems highlights the genomic capacity of these specific strains to synthesize novel reduced polyene scaffolds.

### 3.3. Antibacterial Activity Against Antibiotic-Resistant Pathogens

**Figure 4:**
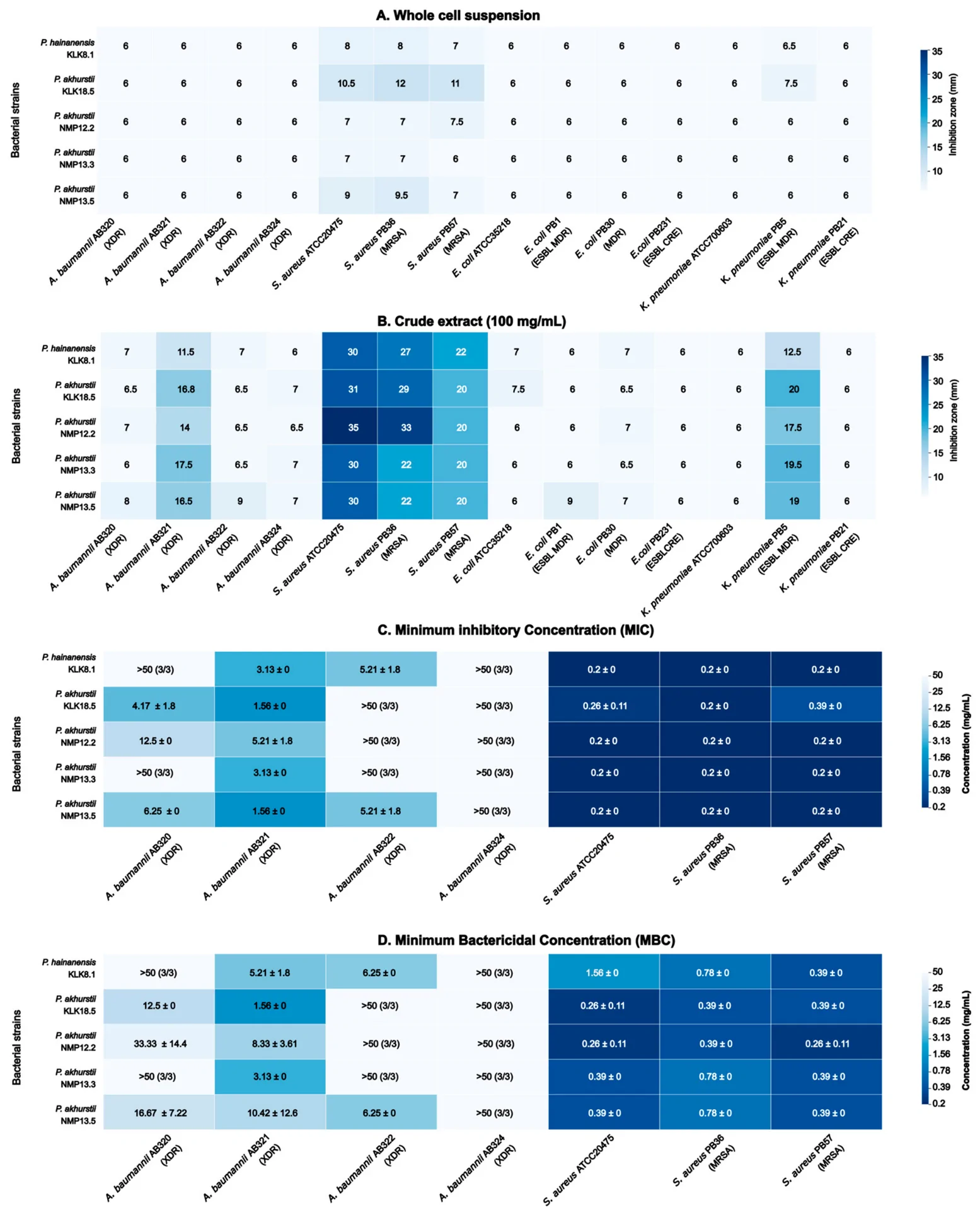
Heatmaps illustrating antibacterial activity of *Photorhabdus* whole-cell suspensions (**A**) and crude extracts (**B**) against 14 pathogenic bacteria. Values are inhibition zone diameters (mm). MIC (**C**) and MBC (**D**) heatmaps of crude extracts. MIC and MBC values are shown as means ± sample standard deviations (SD) in mg/mL; >50 indicates that no endpoint was detected within the tested concentration range. Darker blue represents greater potency. Pathogen abbreviations: AB320, AB321, AB322, and AB324: Extensively drug-resistant (XDR) *Acinetobacter baumannii*; ATCC 20475: *Staphylococcus aureus* ATCC 20475; PB36/PB57 (MRSA): Methicillin-resistant *S. aureus*; ATCC 35218: *Escherichia coli* ATCC 35218; PB30: Multidrug-resistant (MDR) *E. coli*; PB1: ESBL-producing MDR *E. coli*; PB231: ESBL- and carbapenem-resistant (ESBL + CRE) *E. coli*; ATCC 700603: *Klebsiella pneumoniae* ATCC 700603; PB5: ESBL-producing MDR *K. pneumoniae*; KP_PB21: ESBL + CRE *K. pneumoniae*.

To further investigate their antibacterial activity, we evaluated the five *Photorhabdus* isolates in vitro against a panel of antibiotic-resistant pathogens. Disk diffusion assays revealed that whole-cell suspensions exhibited limited antibacterial activity, producing weak inhibition zones (≤10 mm) exclusively against Gram-positive pathogens, including *Staphylococcus aureus* (Figure 4A), with no detectable inhibition against Gram-negative species.

In contrast, the ethyl acetate crude extracts (100 mg/mL) displayed potent antibacterial activity. The average inhibition zone against *S. aureus* exceeded 25 mm, and selective inhibitory activity was additionally observed against the Gram-negative pathogen *Acinetobacter baumannii* AB321 (Figure 4B).

Quantitative evaluation via minimum inhibitory concentration (MIC) (Figure 4C) and minimum bactericidal concentration (MBC) (Figure 4D) assays further corroborated these findings. MIC and MBC values within the tested concentration range were summarized as arithmetic means ± sample standard deviations (SD). Heatmap visualization revealed distinct highly susceptible regions (darker blue) for standard *S. aureus* ATCC20475 and methicillin-resistant *S. aureus* (MRSA) strains PB36 and PB57, demonstrating efficacy at very low concentrations (0.20–0.39 mg/mL). These results indicate that the crude extracts are highly bactericidal against both standard and antibiotic-resistant *Staphylococcus* strains. Conversely, activity against Gram-negative extensively drug-resistant (XDR) *A. baumannii* strains was generally weaker and more variable, reflected by higher MIC and MBC values (Figure 4C,D). This reduced susceptibility is likely driven by the Gram-negative outer membrane, which acts as a robust selective barrier against the uptake of complex bacterial secondary metabolites [46].

## 4. Discussion

Based on whole-genome sequencing and phylogenomic analysis, the five study isolates were classified into two species: strain KLK8.1 as *Photorhabdus hainanensis* and strains KLK18.5, NMP12.2, NMP13.3, and NMP13.5 as *Photorhabdus akhurstii*. KLK8.1 showed genome characteristics comparable to those of the *P. hainanensis* type strain DSM 22397, which was isolated from *Heterorhabditis* sp. in Hainan Island, China, in 1992. DSM 22397 has a 5.50 Mb genome, 43.00% GC content, and 4695 predicted CDS, compared with 5.64 Mb, 43.25% GC content, and 4736 CDS in KLK8.1. DSM 22397 contains 27 BGCs, compared with 29 in KLK8.1. Similarly, the four *P. akhurstii* isolates were compared with DSM 15138, which was isolated from *Heterorhabditis indica* in Grande Terre, Guadeloupe. DSM 15138 has an approximately 5.7 Mb genome, 43% GC content, and 4663 predicted CDS and contains 24 BGCs, compared with 28, 25, 26, and 26 BGCs in KLK18.5, NMP12.2, NMP13.3, and NMP13.5, respectively. These comparisons provide genomic context for the biosynthetic potential of the study isolates.

The identification of a conserved core of biosynthetic gene clusters (BGCs) across these species reflects a strong evolutionary drive to maintain the specialized chemical toolkit required for their complex dual lifestyle as nematode symbionts and insect pathogens [47]. The predicted metabolic profiles (Figure 3) further highlight the critical roles of these core compounds for survival and pathogenesis. For instance, siderophores, including piscibactin and putrebactin, are critical for iron acquisition within the iron-depleted environment of the insect host [7] and have also been reported in *P. akhurstii* bNN168.5_TH, a strain also isolated from Nam Nao National Park, Phetchabun Province, Thailand [27]. Similarly, protease inhibitors such as luminmycin suppress host immune responses [12]. Gamexpeptide [7] likely modulates host immunity to protect the bacterial–nematode partnership and has also been reported as a core compound found across *Photorhabdus* isolates [4,48]. In contrast, some BGCs present in *P. akhurstii* bNN168.5_TH, such as xenocoumacin, were absent from the isolates characterized in this study, highlighting considerable intraspecific variation in the biosynthetic repertoire of *Photorhabdus* isolates. Notably, among the strains examined here, *P. hainanensis* KLK8.1 harbored a unique BGC showing high similarity to chejuenolide [44], a trans-AT PKS reported from the Gram-negative marine bacterial strain *Hahella chejuensis*, distinguishing it from *P. akhurstii*. Similarly, the tilivalline BGC [42] was detected only in *P. akhurstii* strain KLK18.5 among the isolates examined in this study, a pattern also observed in bNN168.5_TH. This distribution suggests that the cluster may have been acquired independently by selected strains through horizontal gene transfer, potentially contributing to differences in their chemical profiles [43].

A major finding of this study is the substantial proportion of the secondary metabolome that remains completely uncharacterized. Approximately 60% of the BGCs showed no similarity to known clusters (Table S3). Likewise, the dominance of COG category S (Function unknown; 25.4–28.4% of all CDS) across all five genomes at the functional annotation level is consistent with this observation, suggesting that a significant fraction of the genomic repertoire of these isolates encodes products of unknown biological roles that extend well beyond secondary metabolism alone. By complementing standard rule-based annotation with predictive, deep-learning-assisted algorithms, we were able to characterize this cryptic landscape and prioritize highly divergent loci that might otherwise evade standard detection. Notably, this integrated approach identified rare, highly reducing type II polyketide synthase (HR-T2PKS) clusters in *P. hainanensis* KLK8.1 and *P. akhurstii* NMP12.2. These clusters, which are structurally distinct from canonical aromatic type II PKSs and are responsible for the biosynthesis of reduced linear polyene compounds [45,49], represent promising targets for future chemical characterization.

Crucially, the biosynthetic potential predicted in silico was accompanied by potent antibacterial activity in vitro (Figure 4). The crude extracts of *P. hainanensis* KLK8.1 and *P. akhurstii* strains KLK18.5 and NMP13.5 exhibited potent antibacterial activity against antibiotic-resistant *Staphylococcus aureus*, indicated by very low MIC and MBC values. Among the isolates, *P. hainanensis* strain KLK8.1 represents a particularly promising candidate for further investigation because of its distinctive genomic features, including the unique presence of the putative chejuenolide pathway and an HR-T2PKS system.

However, we acknowledge certain limitations in the current study. While our integrated genome-mining approach successfully uncovered significant biosynthetic potential, this investigation is currently limited in scope. Furthermore, the potent anti-staphylococcal activity observed in the crude extracts cannot yet be definitively linked to a single, specific BGC. Future studies incorporating a broader, genus-wide pan-genomic analysis, coupled with targeted gene knockouts, heterologous expression, and bioassay-guided chemical fractionation, will be required to physically isolate and structurally characterize the exact novel compounds responsible for this observed bioactivity.

## 5. Conclusions

In conclusion, the genomic and phenotypic characterization of five newly sequenced *Photorhabdus* isolates revealed a highly diverse and largely unexplored set of secondary metabolites. Phylogenomic and ANI analyses classified the isolates into *P. hainanensis* and *P. akhurstii*. Genome mining identified 134 BGCs, of which approximately 60% lacked significant similarity to known gene clusters. Notably, rare highly reducing type II polyketide synthase (HR-T2PKS) pathways were identified, expanding the predicted biosynthetic repertoire of these isolates and highlighting their potential for reduced polyene biosynthesis. The potent antibacterial activity observed in crude extracts, particularly against *S. aureus*, further supports the potential of these isolates for antimicrobial discovery. In particular, the combination of distinctive genomic features and strong antibacterial activity observed for *P. hainanensis* KLK8.1 warrants further investigation to identify and characterize the metabolites responsible for the observed bioactivity.

## Figures and Tables

**Table 1:** Characteristics of isolated strains.

	*P. hainanensis* strain KLK8.1	*P. akhurstii* strain KLK18.5	*P. akhurstii* strain NMP12.2	*P. akhurstii* strain NMP13.3	*P. akhurstii* strain NMP13.5
Closest relative	*P. hainanensis* DSM 22397	*P. akhurstii* DSM 15138	*P. akhurstii* DSM 15138	*P. akhurstii* DSM 15138	*P. akhurstii* DSM 15138
ANI (%)	97.75	97.86	97.87	97.76	97.72
Location	Konglan District, Kamphaeng Phet, Thailand	Konglan District, Kamphaeng Phet, Thailand	Noen Maprang District, Phitsanulok, Thailand	Noen Maprang District, Phitsanulok, Thailand	Noen Maprang District, Phitsanulok, Thailand
EPN host	*H. indica*	*Heterorhabditis* sp.	*H. indica*	*Heterorhabditis* sp.	*H. indica*
Year of isolation	2023	2023	2023	2023	2023
Genome size (Mb)	5.64	5.72	5.85	5.54	5.52
Contigs	16	4	68	25	13
N50 (Mb)	4.69	4.72	0.71	0.96	1.29
GC (%)	43.25	42.81	42.93	42.88	42.85
Completeness (%)	100	100	100	100	100
Contamination (%)	0.58	0.18	4.39	0.08	0.05
Plasmid	Not detected	Not detected	Not detected	Not detected	Not detected
Total BGCs	29	28	25	26	26
Genome accession numbers	SAMN54313145	SAMN54313146	SAMN54313147	SAMN54313148	SAMN54313149

## Data Availability

The raw genomic sequence reads and the assembled genomes, along with accompanying metadata, have been deposited in the National Center for Biotechnology Information (NCBI) under the BioProject accession number PRJNA1393850. The corresponding genome accession numbers are SAMN54313145 (*P. hainanensis* KLK8.1), SAMN54313146 (*P. akhurstii* strain KLK18.5), SAMN54313147 (*P. akhurstii* strain NMP12.2), SAMN54313148 (*P. akhurstii* strain NMP13.3), and SAMN54313149 (*P. akhurstii* strain NMP13.5).

## Supplementary Materials

The following supporting information can be downloaded at https://www.mdpi.com/article/10.3390/microorganisms14102378/s1, Figure S1: COG functional category distribution (%) for five *Photorhabdus* isolates. Bar charts show the percentage of predicted CDS assigned to each COG single-letter category; Table S1: Additional genome statistics for isolated strains; Table S2: Results of dDDH analysis from Type Strain Genome Server (TYGS); Table S3: Summary of biosynthetic gene clusters (BGCs) predicted across all strains using an integrated genome-mining approach.

## Abbreviations

The following abbreviations are used in this manuscript:

ANI	Average nucleotide identity
BGCs	Biosynthetic gene clusters
GCFs	Gene cluster families
HR-T2PKS	Highly reducing type II polyketide synthase
MHA	Mueller–Hinton agar
MBC	Minimum bactericidal concentration
MIC	Minimum inhibitory concentration
ONT	Oxford Nanopore Technologies
