# Supplementary material for: Genomic Analysis and Advanced Mining of Photorhabdus Isolates Reveal Cryptic Polyketide Synthase Clusters and Potent Anti-Staphylococcal Activity
Source: Microorganisms. 2026 Oct 16;14(10):2378. doi: 10.3390/microorganisms14102378 (PMC13707504; doi:10.3390/microorganisms14102378)
Supplement: Supplementary file 1 [file microorganisms-14-02378-s001.zip › microorganisms-4581409-supplementary/Table S1.pdf]

Table S1. Additional genome statistics for isolated strains

	P. hainanensis strain KLK8.1	*P. akhurstii* strain KLK18.5	*P. akhurstii* strain NMP12.2	*P. akhurstii* strain NMP13.3	*P. akhurstii* strain NMP13.5
**Total length**	5,637,198	5,719,030	5,854,085	5,537,670	5,519,192
**Completeness (%)**	100	100	100	100	100
**Contamination (%)**	0.58	0.18	4.39	0.08	0.05
**Number of contigs**	16	4	68	25	13
**Largest contig**	4,690,418	4,717,545	896,602	1,939,730	2,545,520
**CDS**	4,736	4,864	5,147	4,665	4,608
**N50**[1]	4,690,418	4,717,545	705,958	958,776	1,285,792
**N90**[2]	927,000	991,940	175,976	656,392	706,845
**auN**[3]	4,055,099	4,063,491	615,379	1,172,812	1,725,818
**L50**[4]	1	1	4	2	2
**L90**[5]	2	2	10	5	4
**GC (%)**[6]	43.25	42.81	42.93	42.88	42.85
**N's per 100 kbp**[7]	1.24	0.7	21.87	4.15	0.36

[1]**N50:** The length of the contig for which 50% of the genome assembly is contained in contigs of that length or longer. [2]**N90:** The length of the contig for which 90% of the genome assembly is contained in contigs of that length or longer. [3]**auN:** Area under the Nx curve; a continuity metric that accounts for the entire distribution of contig lengths. [4]**L50:** The smallest number of contigs whose combined length makes up at least 50% of the total assembly. [5]**L90:** The smallest number of contigs covering at least 90% of the total assembly length. [6]**GC (%):** Proportion of guanine (G) and cytosine (C) bases in the genome, expressed as a percentage. [7]**N's per 100 kbp:** Number of ambiguous nucleotides ("N") per 100,000 base pairs in the assembly; lower values indicate better completeness.
