# Supplementary material for: Genomic Analysis and Advanced Mining of Photorhabdus Isolates Reveal Cryptic Polyketide Synthase Clusters and Potent Anti-Staphylococcal Activity
Source: Microorganisms. 2026 Oct 16;14(10):2378. doi: 10.3390/microorganisms14102378 (PMC13707504; doi:10.3390/microorganisms14102378)
Supplement: Supplementary file 1 [file microorganisms-14-02378-s001.zip › microorganisms-4581409-supplementary/Table S3.pdf]

**Table S3.** Summary of biosynthetic gene clusters (BGCs) predicted across all strains using an integrated genome-mining approach.

| *P. hainanensis* **strain KLK8.1** | | | | | |
|---|---|---|---|---|---|
| **Cluster** | **Type** | **From** | **To** | **Similarity Confidence** | **Most similar known clusters** |
| **1** | NRPS | 85,552 | 129,943 | - | Unknown |
| **2** | NRPS | 149,462 | 238,592 | High | Kolossin |
| **3** | lassopeptide | 553,454 | 575,965 | - | unknown |
| **4** | NRP-metallophore,NRPS,T1PKS | 576,935 | 639,574 | Low | HTTPCA/prepiscibactin/piscibactin[a] |
| **5** | RRE-containing,resorcinol | 752,537 | 803,693 | - | unknown |
| **6** | amglyccycl | 867,782 | 888,573 | - | unknown |
| **7** | HR-T2PKS | 897,098 | 927,000 | - | unknown |
| **8** | terpene-precursor | 148,154 | 169,071 | - | unknown |
| **9** | NRPS | 188,848 | 232,153 | - | unknown |
| **10** | terpene | 755,392 | 781,033 | High | carotenoid |
| **11** | NRPS | 1,038,872 | 1,075,242 | High | ririwpeptide |
| **12** | NRPS | 1,076,316 | 1,112,711 | - | unknown |
| **13** | betalactone | 1,378,973 | 1,404,472 | High | IOC |
| **14** | T1PKS | 1,488,032 | 1,533,218 | - | unknown |
| **15** | NRPS | 1,639,040 | 1,694,651 | Medium | gamexpeptide A/gamexpeptide B/gamexpeptide E/luminmide B/luminmide D/luminmide E/luminmide F/luminmide G |
| **16** | NRPS | 1,750,625 | 1,813,343 | - | unknown |
| **17** | NRPS | 1,839,130 | 1,889,050 | - | unknown |
| **18** | CDPS | 1,963,905 | 1,984,165 | High | CDPS |
| **19** | PpyS-KS | 2,064,887 | 2,085,936 | - | unknown |

| | | | | | |
|---|---|---|---|---|---|
| **20** | RiPP-like | 2,207,025 | 2,217,936 | - | unknown |
| **21** | NI-siderophore | 2,599,788 | 2,642,254 | High | putrebactin/avaroferrin |
| **22** | leupeptin | 3,005,819 | 3,031,575 | High | leupeptin A/leupeptin B/leupeptin D/leupeptin C |
| **23** | T1PKS,NRPS,NRPS-like,betalactone | 3,035,803 | 3,111,355 | - | unknown |
| **24** | terpene | 3,275,159 | 3,296,253 | - | unknown |
| **25** | NRPS | 3,310,004 | 3,363,063 | Medium | mevalagmapeptide A/mevalagmapeptide B/mevalagmapeptide C/mevalagmapeptide D |
| **26** | transAT-PKS,NRPS | 3,581,027 | 3,645,498 | Low | chejuenolide A/chejuenolide B[a] |
| **27** | NRPS | 3,736,860 | 3,781,857 | - | unknown |
| **28** | azole-containing-RiPP | 4,213,845 | 4,239,945 | - | unknown |
| **29** | NRPS,T1PKS | 4,448,804 | 4,505,774 | Medium | luminmycin A/glidobactin A/cepafungin |

| ***P. akhurstii* strain KLK18.5** | | | | | |
|---|---|---|---|---|---|
| **Cluster** | **Type** | **From** | **To** | **Similarity Confidence** | **Most similar known clusters** |
| **1** | RiPP-like | 69,791 | 80,702 | - | unknown |
| **2** | PpyS-KS | 224,589 | 245,638 | - | unknown |
| **3** | CDPS | 326,100 | 363,153 | High | CDPS |
| **4** | terpene | 653,215 | 678,857 | High | carotenoid |
| **5** | NRPS | 1,141,314 | 1,194,373 | Medium | mevalagmapeptide |
| **6** | crocagin | 1,396,011 | 1,419,100 | - | unknown |
| **7** | NRPS | 1,439,214 | 1,494,509 | - | unknown |

| | | | | | |
|---|---|---|---|---|---|
| **8** | azole-containing-RiPP | 1,918,937 | 1,945,038 | - | unknown |
| **9** | NRPS,T1PKS | 2,162,745 | 2,219,676 | Medium | luminmycin A/glidobactin A/cepafungin |
| **10** | arylpolyene | 2,416,366 | 2,457,550 | - | unknown |
| **11** | resorcinol,NRPS-like | 2,566,200 | 2,630,800 | - | unknown |
| **12** | NRP-metallophore,NRPS,T1PKS | 2,723,343 | 2,786,009 | Low | HTTPCA/prepiscibactin/piscibactin[a] |
| **13** | NRPS | 3,166,642 | 3,255,754 | High | kolossin |
| **14** | NRPS | 3,274,739 | 3,319,130 | - | unknown |
| **15** | terpene-precursor | 3,566,164 | 3,587,081 | - | unknown |
| **16** | NRPS | 3,647,556 | 3,690,861 | - | unknown |
| **17** | T1PKS,NRPS-like,NRPS,leupeptin | 3,916,409 | 4,010,251 | - | unknown |
| **18** | leupeptin | 4,010,345 | 4,033,300 | High | leupeptin A/leupeptin B/leupeptin D/leupeptin C |
| **19** | NRPS | 4,316,121 | 4,360,515 | Low | tilivalline/9-deoxy tilivalline/dihydroxy tilivalline/dehydro tilivalline/dihydroxy-dehydro tilivalline[a] |
| **20** | NI-siderophore | 4,383,706 | 4,426,178 | High | putrebactin/avaroferrin |
| **21** | betalactone | 65,537 | 91,036 | High | IOC |
| **22** | NRPS | 357,602 | 394,006 | - | unknown |
| **23** | NRPS | 395,081 | 411,451 | High | ririwpeptide |
| **24** | NRPS | 437,974 | 487,894 | - | unknown |
| **25** | NRPS | 525,395 | 588,089 | - | unknown |
| **26** | NRPS | 645,834 | 681,475 | Medium | gamexpeptide A/gamexpeptide B/gamexpeptide E/luminmide B/luminmide D/luminmide E/luminmide F/luminmide G |

| | | | | | |
|---|---|---|---|---|---|
| **27** | NRPS | 712,446 | 723,144 | - | unknown |
| **28** | T1PKS | 927,202 | 972,379 | - | unknown |

| ***P. akhurstii* strain NMP12.2** | | | | | |
|---|---|---|---|---|---|
| **Cluster** | **Type** | **From** | **To** | **Similarity Confidence** | **Most similar known clusters** |
| **1** | NRPS | 197,559 | 252,610 | - | unknown |
| **2** | NRPS,NRPS-like | 1 | 38,958 | - | unknown |
| **3** | NRPS | 67,923 | 118,002 | - | unknown |
| **4** | NRPS | 183,078 | 238,719 | Medium | gamexpeptide A/gamexpeptide B/gamexpeptide E/luminmide B/luminmide D/luminmide E/luminmide F/luminmide G |
| **5** | T1PKS | 434,362 | 466,104 | - | unknown |
| **6** | NRPS | 166,090 | 220,686 | - | unknown |
| **7** | terpene,RiPP-like | 746,513 | 783,645 | High | carotenoid |
| **8** | RiPP-like | 749,181 | 760,092 | - | unknown |
| **9** | NRPS | 527 | 103,958 | - | unknown |
| **10** | T1PKS,NRPS-like,NRPS | 119,607 | 195,158 | - | unknown |
| **11** | leupeptin | 209,615 | 235,373 | High | leupeptin A/leupeptin B/leupeptin D/leupeptin C |
| **12** | NRP-metallophore,NRPS,T1PKS,NRPS-like | 32,289 | 97,976 | Low | HTTPCA/prepiscibactin/piscibactin[a] |
| **13** | NRPS | 458,297 | 545,616 | - | unknown |
| **14** | NRPS | 564,824 | 609,215 | - | unknown |
| **15** | betalactone | 103,382 | 130,476 | High | IOC |
| **16** | NI-siderophore | 321,711 | 364,190 | High | putrebactin/avaroferrin |

| | | | | | |
|---|---|---|---|---|---|
| **17** | RiPP-like | 749,181 | 760,092 | - | unknown |
| **18** | PpyS-KS | 872,357 | 893,406 | - | unknown |
| **19** | CDPS | 38,098 | 73,567 | High | CDPS |
| **20** | resorcinol | 88,352 | 129,500 | - | unknown |
| **21** | NRPS,T1PKS | 443,261 | 501,763 | Medium | luminmycin A/glidobactin A/cepafungin |
| **22** | HR-T2PKS | 208,194 | 249,378 | - | unknown |
| **23** | azole-containing-RiPP | 723,709 | 749,810 | - | unknown |
| **24** | NRPS | 118,694 | 161,999 | - | unknown |
| **25** | terpene-precursor | 225,924 | 246,841 | - | unknown |

| ***P. akhurstii* strain NMP13.3** | | | | | |
|---|---|---|---|---|---|
| **Cluster** | **Type** | **From** | **To** | **Similarity Confidence** | **Most similar known clusters** |
| **1** | betalactone | 101,508 | 127,007 | High | IOC |
| **2** | T1PKS | 211,993 | 257,194 | - | unknown |
| **3** | NRPS | 434,264 | 489,905 | Medium | gamexpeptide A/gamexpeptide B/gamexpeptide E/luminmide B/luminmide D/luminmide E/luminmide F/luminmide G |
| **4** | NRPS | 556,726 | 619,420 | - | unknown |
| **5** | NRPS | 648,113 | 678,051 | - | unknown |
| **6** | NRPS | 220,408 | 273,467 | - | unknown |
| **7** | T1PKS,NRPS-like,NRPS | 113,555 | 189,087 | - | unknown |
| **8** | leupeptin | 192,345 | 218,103 | High | leupeptin A/leupeptin B/leupeptin D/leupeptin C |
| **9** | NI-siderophore | 570,740 | 613,337 | High | putrebactin/avaroferrin |
| **10** | RiPP-like | 994,938 | 1,005,849 | - | unknown |
| **11** | PpyS-KS | 1,123,740 | 1,144,789 | - | unknown |

| | | | | | |
|---|---|---|---|---|---|
| **12** | CDPS | 1,227,787 | 1,264,840 | High | CDPS |
| **13** | azole-containing-RiPP | 1,511,325 | 1,537,432 | - | unknown |
| **14** | NRPS,T1PKS | 1,755,060 | 1,811,991 | Medium | luminmycin A/glidobactin A/cepafungin |
| **15** | terpene | 236,873 | 262,519 | High | carotenoid |
| **16** | NRPS | 87,123 | 131,514 | - | unknown |
| **17** | NRPS | 155,207 | 244,319 | High | kolossin |
| **18** | NRP-metallophore,NRPS,T1PKS | 580,605 | 643,271 | Low | HTTPCA/prepiscibactin/piscibactin[a] |
| **19** | resorcinol | 750,745 | 791,893 | - | unknown |
| **20** | arylpolyene | 861,242 | 902,426 | - | unknown |
| **21** | NRPS | 49,742 | 94,739 | - | unknown |
| **22** | NRPS | 494,247 | 550,645 | - | unknown |
| **23** | NRPS | 551,719 | 568,089 | High | ririwpeptide |
| **24** | NRPS | 569,370 | 592,145 | - | unknown |
| **25** | terpene-precursor | 691,288 | 712,205 | - | unknown |
| **26** | NRPS | 734,058 | 777,384 | - | unknown |

| ***P. akhurstii* strain NMP13.5** | | | | | |
|---|---|---|---|---|---|
| **Cluster** | **Type** | **From** | **To** | **Similarity Confidence** | **Most similar known clusters** |
| **1** | terpene-precursor | 153,766 | 174,683 | - | unknown |
| **2** | NRPS | 196,555 | 239,881 | - | unknown |
| **3** | T1PKS,NRPS-like,NRPS | 471,813 | 547,345 | - | unknown |
| **4** | leupeptin | 550,603 | 576,361 | High | leupeptin A/leupeptin B/leupeptin D/leupeptin C |
| **5** | NI-siderophore | 927,716 | 970,313 | High | putrebactin/avaroferrin |

| | | | | | |
|---|---|---|---|---|---|
| **6** | NRPS | 91,716 | 136,107 | Low | unknown |
| **7** | NRPS | 159,800 | 248,912 | High | kolossin |
| **8** | NRP-metallophore,NRPS,T1PKS | 583,634 | 646,300 | Low | HTTPCA/prepiscibactin/piscibactin[a] |
| **9** | resorcinol | 753,034 | 794,182 | - | unknown |
| **10** | arylpolyene | 863,529 | 904,713 | - | unknown |
| **11** | NRPS,T1PKS | 1,093,553 | 1,150,484 | Medium | luminmycin A/glidobactin A/cepafungin |
| **12** | azole-containing-RiPP | 1,368,111 | 1,394,218 | - | unknown |
| **13** | NRPS | 1,818,444 | 1,863,441 | - | unknown |
| **14** | NRPS | 2,138,363 | 2,191,422 | - | unknown |
| **15** | RiPP-like | 51,043 | 61,954 | - | unknown |
| **16** | PpyS-KS | 179,845 | 200,894 | - | unknown |
| **17** | CDPS | 283,892 | 320,945 | High | CDPS |
| **18** | terpene | 548,509 | 574,155 | High | carotenoid |
| **19** | NRPS | 7,247 | 43,645 | - | unknown |
| **20** | NRPS | 44,719 | 61,089 | High | ririwpeptide |
| **21** | NRPS | 62,370 | 85,145 | - | unknown |
| **22** | NRPS | 112,256 | 122,176 | - | unknown |
| **23** | NRPS | 170,869 | 233,563 | - | unknown |
| **24** | NRPS | 300,384 | 356,025 | Medium | gamexpeptide A/gamexpeptide B/gamexpeptide E/luminmide B/luminmide D/luminmide E/luminmide F/luminmide G |
| **25** | T1PKS | 533,132 | 578,333 | - | unknown |
| **26** | betalactone | 663,510 | 689,009 | High | IOC |

[a] Putative identification confirmed via manual verification of PKS/NRPS domain architecture against MIBiG references, despite low automated similarity score.
